# A pattern of peri‐implantitis affecting middle implants in 3‐implant splinted prostheses

**DOI:** 10.1002/cap.10274

**Published:** 2023-11-27

**Authors:** Braedan RJ Prete, Michael AJ Silva, Brian C. Wong, Douglas A. Deporter

**Affiliations:** ^1^ Graduate Periodontics Faculty of Dentistry University of Toronto Toronto Canada; ^2^ Discipline of Periodontology Faculty of Dentistry University of Toronto Toronto Canada

**Keywords:** bone loss, case reports, dental implant, implant‐supported dental prostheses, peri‐implantitis, splinted crowns

## Abstract

**Background:**

Previous investigators have noted an increased risk of crestal bone loss and failure of the middle implant of 3‐implant‐splinted (3‐IS) fixed dental prostheses (FDPs). Possible causes have included ill‐fitting prostheses, unhygienic prosthetic contours, and discrepancies in prosthetic platform heights.

**Methods & Results:**

We identified four cases in which the middle implant of a 3‐IS multiunit FDP suffered advanced bone loss, ultimately leading to implant removal. While more than one possible risk for implant failure existed in each case, a common thread was that the prosthetic platform of the middle implant for all patients was coronally positioned relative to the corresponding mesial and/or distal implants.

**Conclusions:**

Splinting three adjacent implants into one prosthesis may add risk for a variety of reasons possibly including small differences in the heights of the three prosthetic tables.

**Key points:**

**Why are these cases new information?**

- Our observations suggest that discrepancies between implant prosthetic platforms supporting 3‐implant splinted, multiunit FDPs may be an added risk factor for middle implant failure.

**What are the keys to successful management of these cases?**

- It is possible that small differences in apico‐coronal implant positioning with 3‐implant splinted multiunit FDPs may affect the success of the middle implants.

**What are the primary limitations to success in these cases?**

- There is limited literature involving precise protocols and long‐term outcomes of 3‐implant splinted implant restorations.
- Studies comparing 3‐implant splinted FDPs to other configurations are needed.

## INTRODUCTION

There is a dizzying array of possible risk factors for marginal bone loss and subsequent failure of endosseous, threaded dental implants often making it difficult to determine the dominant one(s) in a particular instance. Smoking is a major cause,^1^ as is a history of periodontitis,^2^ but issues as basic as minimal peri‐implant keratinized soft tissue width and thickness, improper 3D implant positioning, and depth of placement relative to buccal bone crest will all have a role to play.^3^, ^4^, ^5^ Prosthetic design will also contribute if the implant‐supported fixed dental prosthesis (IS‐FDP) is not passively fitted^6^, ^7^ and/or if emergence profiles of individual restorations, including pontics, are not suitably designed for thorough daily homecare and routine professional maintenance,^8^, ^9^, ^10^ or if loss of proximal contact with adjacent teeth develops.^11^

Splinting adjacent implants has been linked to crestal bone loss and peri‐implantitis by some authors,^12^, ^13^ but not others,^14^ although this would likely be affected in each case by prosthesis fit and design. Yi et al.^9^ reported that implants splinted between two other implants (SP middle group) in a single prosthesis had a high risk of peri‐implantitis, although this varied with the emergence profile. The highest risk was associated with convex profiles. This study also noted a 4.66‐fold higher risk of peri‐implantitis in the SP middle group compared with singly‐restored implants.^9^ In a subsequent report, the same authors provided an assessment of biological complications related to splinting over 15 years for 888 implants in 423 patients.^15^ Complications had affected 26.4% of nonsplinted vs. 45.4% of splinted implants, and again the SP middle group had the highest risk of peri‐implant disease.

Another recent study suggested another possible risk factor for bone loss with splinted prostheses by suggesting that minimal vertical discrepancies between prosthetic platforms of splinted adjacent implants might contribute.^16^ In the present case series, we have explored this last possible risk factor with failing middle implants in splinted IS‐FPDs.

## MATERIALS AND METHODS

We identified four cases in which the middle implant of a 3‐IS multiunit FDP demonstrated advanced bone loss requiring eventual explantation. All are presented in a case report style. Patients were selected based on patterns observed during routine posttreatment follow‐up. Patient 1 had been treated in a private practice setting, while the other patients were seen at the Graduate Periodontics Clinic at the Faculty of Dentistry, University of Toronto. Consent was obtained from all patients prior to the writing of this manuscript.

### Clinical scenarios

#### Case report one

**FIGURE 1:**
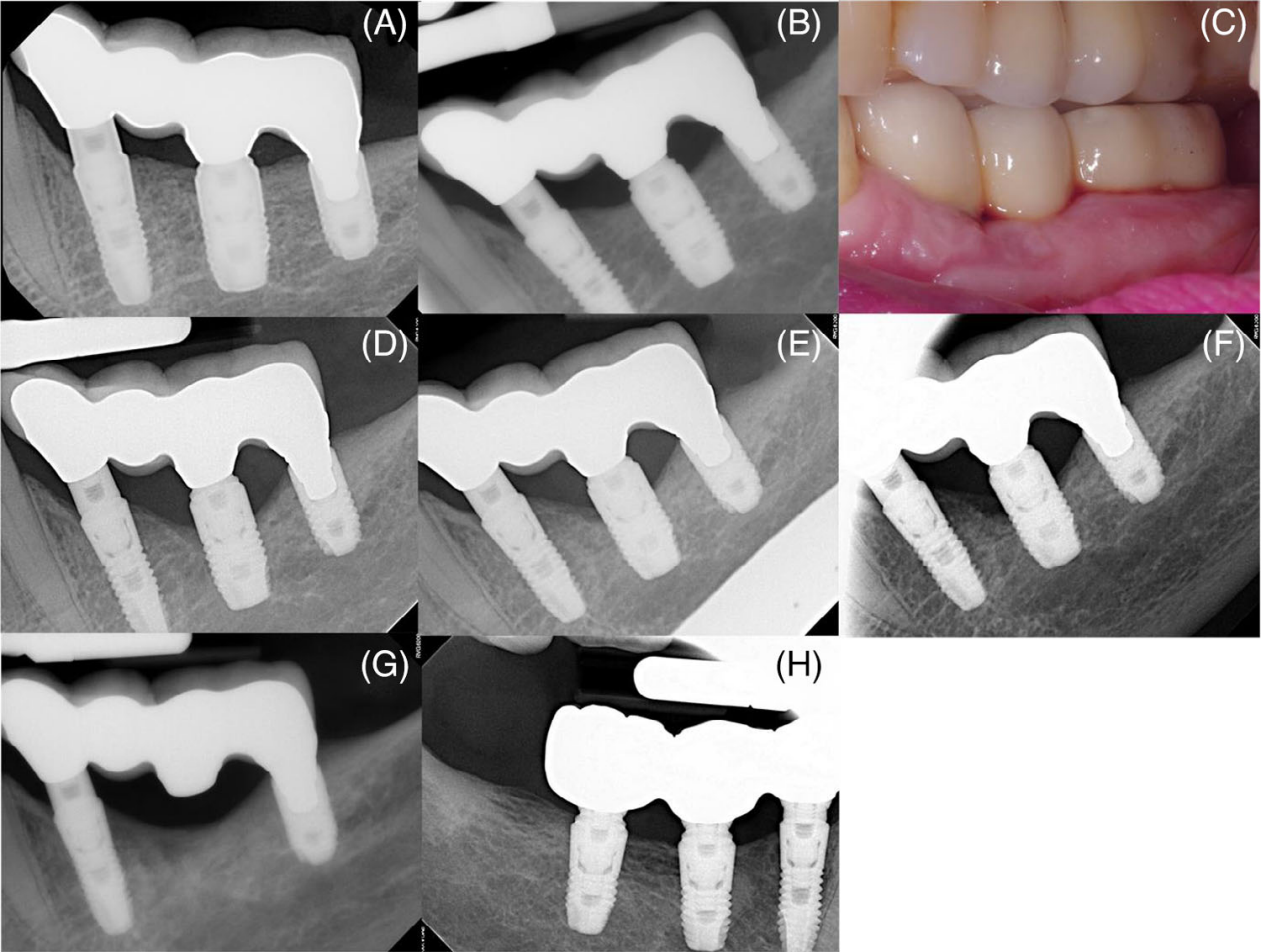
Radiographic series and one clinical photograph for patient 1. (A) A 3‐IS four‐unit FDP was placed in the mandibular left posterior sextant in 2016 (Astra Tech OsseoSpeed TX); (B) early signs of peri‐implant bone loss were suspected in 2018; (C) clinically the implants appeared healthy; after subsequent follow‐up in (D) 2019, (E) 2020, and (F) 2021, the middle implant was determined to be hopeless, was explanted, and the modified FDP re‐inserted; (G) at 3‐years, the modified FDP in the mandibular left posterior sextant and (H) the 3‐IS three‐unit FDP in the mandibular right posterior sextant both appear radiographically stable.

Patient 1 was a 78‐year‐old female never‐smoker with a history of treated periodontitis (Stage III, Grade B, generalized).^17^ Her medical history included hypertension, hypercholesterolemia, and gastroesophageal reflux disease (GERD), respectively, controlled with Amlodipine, Rosuvastatin, and Pantoprazole. In 2016, a 3‐IS four‐unit FDP was placed in quadrant 3 (Figure 1A) and a 3‐IS three‐unit FDP in quadrant 4, both of which were placed on three splinted implants (Astra Tech OsseoSpeed TX). In 2018, early signs of peri‐implant bone loss were noted at the middle implant of the IS‐FPD in quadrant 3 (Figure 1B). Clinically, there was bleeding with gentle probing possibly related to the prosthesis design (Figure 1C). The implant platforms of both the middle and distal implants were positioned apically with respect to the mesial implant, and the middle implant was positioned slightly coronal to the distal implant. After subsequent follow‐up visits between 2019 and 2021 (Figures 1D–F), the prognosis of the middle implant was determined to be hopeless. Interestingly, the IS‐FDP in quadrant 4 continued to remain healthy (Figure 1G). As seen in Figure 1H, the middle implant platform of the healthy prosthesis was positioned apically with respect to both the mesial and distal implants.

#### Case report two

**FIGURE 2:**
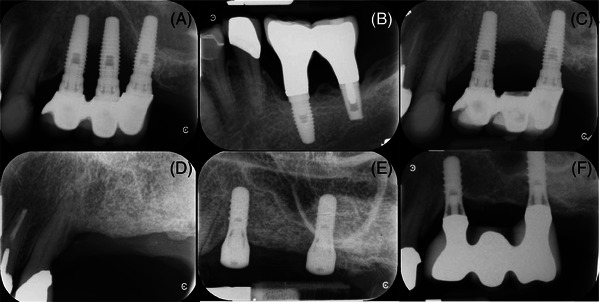
Radiographic series for patient 2. (A) In 2014, 20 years after placement, the patient presented with an ailing 3‐IS three‐unit FDP; (B) the opposing 2‐IS three‐unit FDP (Nobel Replace CC, Endopore) was healthy; (C) the middle implant of the 3‐IS three‐unit FDP was removed while severe bone loss at the mesial and distal implants also were later removed (D); (E) after GBR, two implants (Straumann SP) were placed; (F) 3‐year recall of the retreatment.

The second patient was a 62‐year‐old female who presented in 2014 with a failing cement‐retained 3‐IS three‐unit FDP (Nobel Replace CC) (Figure 2A). These implants had been placed by her dentist in private practice in 1994. The patient'smedical history included smoking until 2001, hypertension controlled with Perindopril 2 mg, a history of a transient ischemic attack, daily aspirin 81 mg, and osteopenia controlled with Risedronate 35 mg p.o. weekly. The oral bisphosphonate had been used for 1 year in 2012 and then discontinued. The patient admitted to irregular implant maintenance from the time of placement until her presentation in 2014.

As seen in figure 2A, the middle implant had lost >50% of its bony support with apparent extension to the approximating surfaces of the other two implants. As with patient 1, the prosthetic platform of the middle implant was slightly coronal to its adjacent implants. Furthermore, the angulations of the middle and distal implants were non‐parallel. The opposing prosthesis was a 2‐implant splinted (2‐IS) FDP in apparent good health (Figure 2B). The degree of destruction to the remaining two implants in the maxilla was severe (Figure 2C), and ultimately led to their removal. A GBR procedure was performed with direct sinus elevation (Figure 2D), followed by a free‐gingival graft to augment the peri‐implant keratinized tissues with ultimate placement of two tissue level implants^‖^ later restored with a 2‐IS three‐unit FDP (Figures 2E–F).

#### Case report three

**FIGURE 3:**
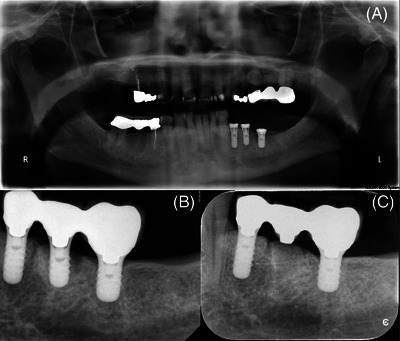
Radiographic series for patient 3. (A) a panoramic image showing the three implants (Straumann SP) placed at the left mandibular posterior sextant in 2011; (B) in January 2023, the middle implant was seen to have suffered severe bone loss and was considered a failure; (C) it was removed, its socket grafted and the modified FDP re‐inserted.

Patient 3 was a medically fit and periodontally healthy 60‐year‐old male who received three implants (Straumann SP) restored with a screw‐retained 3‐IS three‐unit FDP in his left mandibular posterior sextant. The implants were placed in 2011 (Figure 3A). Prior to treatment, he had smoked 2 packs/day for 40 years but had ceased in 2010 to receive the implants. In 2023, he presented with severe generalized attrition and reported a history of bruxism. The middle implant showed advanced bone loss, while the mesial and distal implants showed stable radiographic bone levels (Figures 3A, B). The prosthetic platform of the middle implant was noticeably coronal to that of the distal implant (Figure 3C). The decision was made to remove the screw‐retained prosthesis, extract the problem implant, graft the middle implant socket, and re‐insert the FDP (Figure 3D).

#### Case report four

**FIGURE 4:**
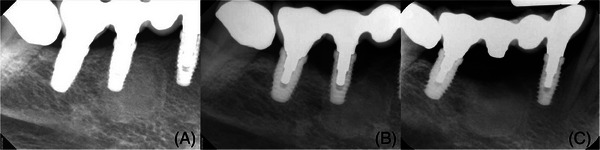
Radiographic series for patient 4. (A) a periapical radiograph taken at the time of prosthesis installation in 2020; (B) in 2022, the middle implant showed advanced horizontal, circumferential crestal bone loss and was removed and the outcome appeared stable one year later (C).

Patient 4 was a 56‐year‐old, nonsmoking female originally diagnosed with periodontitis (Stage III, Grade B, generalized)^17^ wishing to replace her right mandibular canine, first and second bicuspids and first molar with an implant‐supported FDP (Biomet 3i T3 Tapered). She had been using Synthroid for many years and Prolia for the previous 6 months. Radiographically, there was a dense bone island in the edentulous bicuspid region (Figure 4A). After successful periodontal disease management, a 3‐IS four‐unit FDP was provided in 2020 with the middle implant partially embedded in the dense bone irregularity. To encourage re‐vascularization here, the affected osteotomy had received a platelet‐rich fibrin preparation^18^ immediately before implant insertion.

Due to the pandemic, after restoration the patient was not seen again until 2022, at which time the middle implant clearly had suffered extensive bone loss (Figure 4B). It was noted that the prosthetic platform of this implant was visibly coronal to that of the distal implant. Surgical exposure of the site confirmed that the middle implant was hopeless with advanced horizontal bone loss. This implant was removed without bone grafting of the defect and the modified FDP re‐inserted (Figure 4C) after complete soft tissue healing.

## DISCUSSION

It is common practice for clinicians to splint dental implants to contain costs and minimize mechanical complications with FDPs over time. However, splinting requires meticulous planning and prosthetic design with delivery of a prosthesis that is well‐fitting, easily cleansable, ideally screw‐retained and equilibrated to minimize risk of occlusal overload. Perhaps as a result, splinted implants do show higher risk of biological complications than their non‐splinted counterparts. Accordingly, at least one group of investigators has recommended that where three or more adjacent implants are placed, the best approach may be to restore them as individual single units.^19^

In their retrospective study over 15 years, Yi et al.^15^ reported that the middle implant of 3‐IS FDPs showed a greater risk of crestal bone loss than the corresponding mesial and distal implants. To help to explain these findings, Lin et al.^16^ studied whether small discrepancies in vertical prosthetic platform heights of adjacent splinted implants could affect crestal bone loss. Retrospective radiographic data for 337 implants in 156 patients with two or three adjacent standard length and diameter implants restored either singly or as splinted units were examined. Vertical platform discrepancies between adjacent implants and associated proximal bone loss were measured with computer software. Of concern was the finding that 2‐ or 3‐IS FDPs with vertical discrepancies of ≥0.5 mm showed greater bone loss than nonsplinted adjacent implant restorations. Indeed, most 3‐IS prostheses with these discrepancies had at least one implant with ≥1 mm bone loss, and in 90.9% of the cases, the most apically positioned implant demonstrated the least degree of peri‐implant bone loss.

The four patients reported here each certainly had more than one possible contributing factor for the observed severe bone loss at their middle implants. Due to the COVID‐19 pandemic, all patients had some degree of poor implant maintenance care, but for patient 2, this had been the case for 20 years. Patients 1 and 4 had a history of periodontitis, a well‐documented risk factor for peri‐implantitis.^20^, ^21^ Furthermore, patient 1 had been using a proton‐pump inhibitor (PPI) for several years, PPIs being another risk factor for implant failure.^22^, ^23^ Patient 3 was a heavy bruxer with generalized severe attrition, bruxism being a high risk for implant failure as well.^24^, ^25^ Patient 4 had an existing dense bone island which may have compromised the integration of her middle implant. Furthermore, she had been using Prolia for osteopenia starting 6 months before implant surgery and had a history of treated chronic, moderately severe periodontitis.

The 3‐IS FDPs documented here all had vertical radiographic discrepancies in their prosthetic platforms with those of the middle implants generally being coronal to either or both of their mesial and/or distal counterparts. Following the findings of Lin et al.,^16^ this suggested to us that subtle differences in heights of prosthetic platforms and/or differences in implant parallelism may put unfavorable stresses on the middle implants. In keeping with this speculation, it was noted that the one successful 3‐IS FDP in patient 1 had its middle implant positioned apical to its adjacent implants. Remember that Lin^16^ found that the most apically‐positioned implant of those in 3‐IS FDPs had the least bone loss. Although this is pure speculation currently, our intention is to undertake further studies to explore this hypothesis.

## CONCLUSION

We have identified four cases where the middle implant of 3‐IS FDPs failed. A common finding was an apico‐coronal discrepancy of the middle implant prosthetic platform relative to its mesial and/or distal implants. It may be that this nonmatching of implant platform levels can lead to unfavorable stresses for crestal bone of middle implants.

## AUTHOR CONTRIBUTIONS

All authors contributed to the realization of this paper and approved the manuscript before submission. Braedan RJ Prete: literature search, original drafts, and final review and editing. Brian C. Wong: literature search and original drafts. Michael AJ Silva literature search and original drafts. Douglas A. Deporter: conceptualization, methodology, interpretation, and final review and editing.

## CONFLICT OF INTEREST STATEMENT

The authors declare no conflict of interest.
